# YouTube as a source for clavicle fracture education: a quality, comprehensibility, and reliability assessment

**DOI:** 10.1016/j.xrrt.2026.100800

**Published:** 2026-06-19

**Authors:** Kirk Terada-Herzer, Ryan Nishi, Tyler Shimabukuro, Kyle Obana, Julian Rimm, Kyle Ishikawa, Matthew Nishimura, Joanna Kim, Lorrin Lee

**Affiliations:** a John A. Burns School of Medicine, University of Hawaii at Manoa, Honolulu, HI, USA; b Department of Orthopedic Surgery, Columbia University Irving Medical Center, New York, NY, USA; c Department of Orthopedic Surgery, University of Hawaii, Honolulu, HI, USA

**Keywords:** Clavicle fracture, YouTube, Educational quality, Patient information, Video reliability, Orthopedic trauma

## Abstract

**Background:**

Clavicle fractures are common injuries, and patients frequently seek supplemental information online. YouTube is widely used for health-related content, but the quality and completeness of information related to clavicle fractures are not well defined. This study evaluated the reliability, comprehensibility, and educational completeness of clavicle fracture videos on YouTube.

**Methods:**

A systematic search of YouTube was performed using 4 clavicle fracture–related terms. After screening for eligibility, the 50 most-viewed English-language videos were analyzed. Videos were categorized by source and content type. Reliability was assessed using the *Journal of the American Medical Association* (JAMA) Benchmark Criteria; comprehensibility was rated on a 4-point scale; and educational completeness was evaluated using an 18-point Clavicle Fracture Rating (CFR). Two independent reviewers scored all videos. Nonparametric analyses, including Kruskal–Wallis tests and Wilcoxon rank-sum tests with Holm correction, were applied.

**Results:**

The median CFR score was 3 (Q1 = 1, Q3 = 6). The mean JAMA score was 1.4 and the mean comprehensibility score was 3.46. Physician-generated videos demonstrated significantly higher JAMA scores than patient-generated videos (*P* < .01), whereas patient-generated videos showed significantly higher comprehensibility (*P* < .01). YouTube “shorts” had significantly lower CFR scores (mean 0.8) than standard-length videos (mean 4.3; *P* < .01). Across all categories, essential elements of clavicle fracture evaluation and management were infrequently addressed.

**Conclusion:**

YouTube videos related to clavicle fractures demonstrated low reliability and limited educational completeness, despite generally favorable comprehensibility. These findings suggest that commonly viewed online resources provide insufficient information for patient education.

Clavicle fractures represent 3–5% of all fractures and occur most frequently in young athletes and older adults prone to falls.^6^^,^^10^^,^^13^^,^^23^ Large epidemiologic registries have shown rising incidence and increasing operative management over the past 2 decades.^2^^,^^11^^,^^12^ Although many clavicle fractures heal without surgery, displaced midshaft fractures carry higher risks of nonunion and symptomatic malunion than previously appreciated.^16^^,^^21^^,^^31^ Randomized trials and meta-analyses have demonstrated that operative fixation can reduce nonunion rates, improve short-term function, and expedite recovery in selected patients.^4^^,^^9^^,^^18^^,^^33^^,^^36^^,^^37^ Functional outcomes, however, vary, and shared decision-making remains essential across treatment options.^15^^,^^20^^,^^28^

Patients increasingly seek supplemental medical information online, and YouTube is among the most frequently accessed websites worldwide.^34^ Prior research across orthopedic subspecialties, including rotator cuff disease, carpal tunnel syndrome, knee arthroplasty, patellar instability, femoroacetabular impingement, trigger finger, and ulnar collateral ligament injuries, consistently demonstrates that YouTube videos often lack reliability, transparency, and clinical accuracy.^1^^,^^5^^,^^7^^,^^14^^,^^17^^,^^27^^,^^32^^,^^35^^,^^36^ Studies evaluating patient information on the internet more broadly echo similar concerns.^25^^,^^26^

Despite the high prevalence of clavicle fractures and their relevance to younger, internet-reliant populations, no prior investigation has systematically evaluated clavicle-specific fracture content on YouTube. Emerging trends toward short-form content further amplify concerns, as algorithm-driven promotional biases may favor brevity over educational completeness.^19^^,^^22^^,^^24^ Understanding the reliability, comprehensibility, and medical completeness of existing online resources is essential, as misinformation can influence patient expectations, anxiety, and perceptions of operative vs. nonoperative management.

The purpose of this study was to evaluate the reliability, comprehensibility, and educational completeness of YouTube videos related to clavicle fractures using standardized scoring tools. We hypothesized that video quality would be low overall and would differ significantly based on video source and content type.

## Materials and methods

### Search strategy and video selection

A systematic search of YouTube was performed on June 17, 2025, using 4 search terms related to clavicle fractures: “clavicle fracture,” “broken clavicle,” “fractured collarbone,” and “broken collarbone.” YouTube is known to display dynamic, algorithm-dependent search results influenced by user behavior and engagement trends.^19^^,^^24^ For each term, the 50 highest-viewed videos were collected, yielding 200 total results. After removal of duplicates, non-English content, and unrelated videos, 98 unique videos remained. From these, the 50 most-viewed were selected to best represent the content most likely encountered by patients seeking online information.^26^^,^^35^

### Video characteristics and categorization

Recorded video information included duration, views, likes, dislikes, upload date, and whether the video was a standard YouTube video or a YouTube “short,” an increasingly promoted short-form format with known limitations in educational depth.^19^^,^^22^ A video power index was calculated as: *(likes/d × views/d)/100*, consistent with prior digital media analyses.^32^

Videos were categorized by source (academics, physicians, health professionals, trainers/physical therapists, medical sources, patients, and commercial). This approach aligns with established YouTube evaluation methodology used in orthopedic and sports medicine research.^1^^,^^7^^,^^14^^,^^27^ Due to low numbers in several categories, commercial, health professional, medical source, and trainer/physical therapist videos were consolidated into an aggregated “other” group for statistical analysis, a common approach in prior YouTube reliability studies.^1^^,^^17^^,^^32^

Videos were also classified by content type: patient experience, disease-specific information, surgical technique, nonsurgical management, exercise training, or advertisement. Low-frequency groups were consolidated into an “other content” category.^17^^,^^27^

### Quality assessment

To ensure consistent results across rating individuals, 5 high-quality videos were selected, rated, and compared independently by authors, KT and RN, prior to the assessment of the entire collection. This calibration phase allowed for the opportunity to create additional criteria for grading and discuss potential points of misinterpretation. Disagreements in final scores were resolved through consensus. Three scoring systems were used to evaluate reliability, comprehensibility, and educational completeness.

1. ***Journal of the American Medical Association* (JAMA) Benchmark Criteria (0–4 points)** — an established tool for assessing source transparency and reliability (Table I).^25^^,^^29^

**Table I:** A description of the 4 benchmarks of the JAMA scoring system used to assess reliability of online health information.

Benchmark criteria	Description
Authorship	Author and contributor affiliations (and their relevant credentials) should be clearly provided.
Attribution	References for all information should be listed clearly with relevant copyright information.
Disclosure	Fully discloses conflicts of interest, advertisements, underwriting, sponsorships, or other funding sources.
Currency	Provides the date the content was created and last updated.

*JAMA*, Journal of the American Medical Association.
2. **Comprehensibility Scale (1–4 points)** — adapted from prior orthopedic YouTube studies evaluating patient-centered communication (Table II).^32^^,^^35^

**Table II:** A description of the 4 criteria that make up the comprehensibility measure, which evaluates the use of medical jargon.

Score	Description
1	Negligible use of medical jargon
2	Minimal use of medical jargon
3	Moderate use of medical jargon
4	Heavy use of medical jargon
3. **Clavicle Fracture Rating (CFR) — 18 points** — a novel scoring system developed for this study based on existing clavicle fracture epidemiology, treatment frameworks, and classification systems (Table III).^3^^,^^8^^,^^12^^,^^23^^,^^30^^,^^33^^,^^38^

**Table III:** Descriptions of 18 characteristics used for the Clavicle Fracture Rating (CFR) with relevant requirements.

Characteristic	Points	Requirements
General information		
Anatomy description	1	Any mention of clavicle anatomy
Clavicle fracture description	1	Any description of a clavicle fracture, including, but not limited to, nondisplaced, displaced, or open clavicle fractures
Risk factors	1	Any mention of clavicle fracture risk factors, including, but not limited to, sports or other high-impact activities
Mechanisms	1	Any mention of how a clavicle fracture can occur
Differential diagnosis	1	Any mention of differential diagnoses for clavicle fractures, such as acromioclavicular joint injury or proximal humerus fracture
Frequency	1	Any mention of prevalence or incidence of clavicle fractures
Demographics	1	Any mention of patient demographics, such as age or sex
Diagnosis		
Fracture location	1	Any mention of where the clavicle fracture occurred (proximal, midshaft, distal)
Additional classification	1	Any mention of a more specific Clavicle Fracture Rating (either Neer or Arbeitsgemeinschaft für Osteosynthesefragen (AO) classifications (Lian; Meinberg))
Physical exam	1	Any mention of pertinent physical exam findings
Radiographic findings	1	Any mention of radiographic findings, such as x-ray appearance
Indications		
Indications for operative treatment	1	Any mention of the indications for operative treatment
Indications for nonoperative treatment	1	Any mention of the indications for nonoperative treatment
Treatment		
Operative treatment	1	Any mention of the nature of operative treatment
Nonoperative treatment	1	Any mention of the nature of nonoperative treatment
Prognosis		
Recovery timeline	1	Any mention of the typical recovery time for operative or nonoperative treatment
Complications	1	Any mention of possible operative or nonoperative complications

### Sex and gender reporting statement

This study analyzed publicly available online videos and included no human subjects or identifiable patient information. Sex and gender characteristics were not present within the dataset and therefore could not be incorporated into analysis. This limitation should be considered when interpreting generalizability.

### Statistical analysis

Normality was assessed using the Shapiro–Wilk test. Because no variable was normally distributed, nonparametric tests were used, consistent with prior YouTube-based orthopedic investigations.^17^^,^^27^ Kruskal–Wallis tests evaluated overall group differences followed by Wilcoxon rank-sum tests for pairwise comparisons with Holm correction for multiple testing. Significance was set at *P* < .05. Analyses were performed using R version 4.5.0.

### Ethical review

All data were obtained from publicly accessible YouTube sources. No patient data or interaction occurred; therefore, this study was exempt from institutional review board review.

## Results

### Video characteristics

The 50 included videos received a combined total of 16.5 million views. Median number of views was 329,567 and the median number of likes was 2,658 (Table IV). The median CFR score across all videos was low, with a median CFR of 3. Neither source category (*P* = .403) or content type (*P* = .169) demonstrated high CFR scores.

**Table IV:** Median values and quartile ranges for various video characteristics.

Characteristic	Median (Q1, Q3)
Duration (minutes)	3.9 (1.7, 9.93)
Number of views	329,567 (175,864, 802,911)
Number of likes	2,658 (1,105, 8,300)
Video power index	1.2 (0.28, 78.8)
CFR score	3 (1, 6)

**Figure 1:**
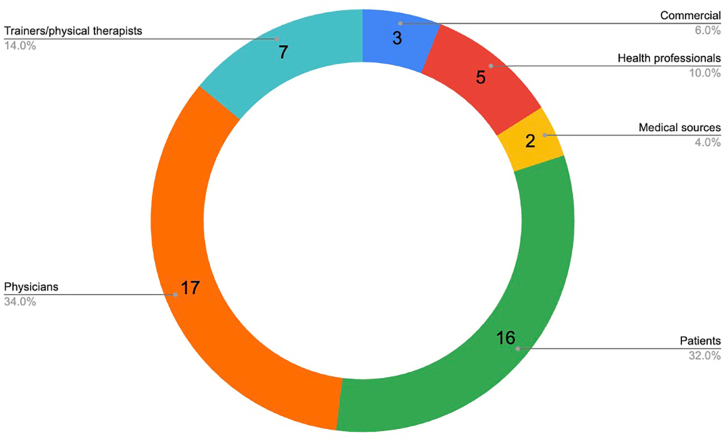
Distribution of clavicle fracture video sources on YouTube, including physicians, patients, and trainers/physical therapists. Percentages reflect each category's share of the total sample (N = 50).

Videos were uploaded by physicians (34%), patients (32%), trainers of physical therapists (14%), and other health professionals (10%) (Fig. 1). After consolidation of low-frequency categories, the statistical analyses compared physicians, patients, academics, and other sources.

**Figure 2:**
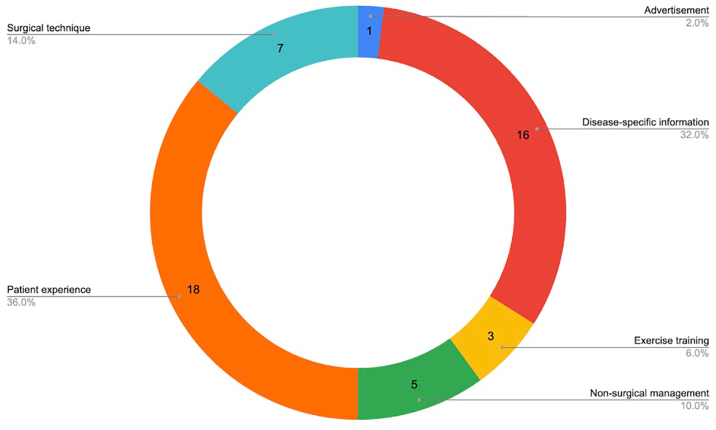
Distribution of clavicle fracture video content on YouTube, including patient experience, disease-specific information, and surgical technique. Percentages reflect each category's share of the total sample (N = 50).

**Figure 3:**
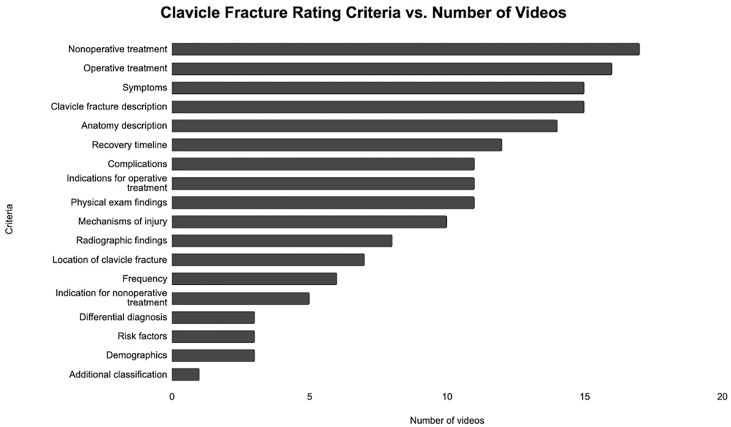
Distribution of videos meeting each predefined Clavicle Fracture Rating criterion, indicating how frequently different aspects of clavicle fracture management and education are addressed across the sample (N = 50).

The most common content types were patient experience (36%) and disease-specific information (32%) (Fig. 2). The criteria most often included in videos were descriptions of treatment for nonoperative management, treatment for surgical management, and general symptoms. The criteria mentioned the least were risk factors, demographics, and an additional classification of the clavicle fracture (Fig. 3).

Thirty-seven (74%) videos received a score of 1 on the JAMA Benchmark Criteria measure, and zero videos satisfied all 4 of the benchmarks (Table V). There was a statistically significant difference between JAMA Benchmark Criteria scores depending on video source. Videos made by physicians scored higher than videos made by patients (1.5 vs. 0.9, *P* = .005). JAMA Benchmark Scores differed significantly across content categories, with patient experience content scoring lower (0.9) than both disease-specific content (1.4, *P* = .005) and “other” content types (1.4, *P* = .019) (Table VI).

**Table V:** JAMA Benchmark scores of including videos, with counts and percentages provided for each score category (N = 50).

JAMA benchmark criteria	Count (%)
1	37 (74%)
2	11 (22%)
3	2 (4%)
4	0 (0%)

*JAMA*, Journal of the American Medical Association.

**Table VI:** Mean values and standard deviations for video power index, JAMA benchmark criteria, video comprehensibility, and total CFR score, divided by video source and content type.

Characteristic	Video power index		JAMA benchmark criteria		Video comprehensibility		Total CFR score	
	N = 50∗	*P* value†	N = 50‡	*P* value†	N = 50§	*P* value†	N = 50‖	*P* value†
Video source		.145		**.005**		**.004**		.403
All others	40,091.31 (164,169.27)		1.3 (0.6)		3.6 (0.6)		3.4 (4.2)	
Patients	829.76 (2,455.02)		0.9 (0.3)		3.9 (0.3)		2.3 (3.1)	
Physicians	19,418.41 (79,359.30)		1.5 (0.6)		2.8 (1.4)		4.4 (4.8)	
Video Content		.346		**.007**		**.006**		.169
All others	20,629.47 (81,799.67)		1.4 (0.7)		3.1 (1.3)		2.4 (2.9)	
Disease-specific information	42,595.64 (169,217.52)		1.4 (0.5)		3.2 (1.0)		5.6 (5.3)	
Patient experience	741.09 (2,320.51)		0.9 (0.2)		3.9 (0.2)		2.2 (3.0)	

*JAMA*, Journal of the American Medical Association; *SD*, standard deviation.

Bold indicates statistically significant *P* values.

∗ Video power index: mean (SD).

† Kruskal-Wallis rank sum test.

‡ JAMA benchmark criteria: mean (SD).

§ Video comprehensibility: mean (SD).

‖ Total: mean (SD).

Differences in CFR among videos categorized as disease-specific information (5.6), patient experience (2.2), and “other” (2.4) were not statistically significant (*P* = .169). There was also no statistically significant difference in mean CFRs for videos made by patients compared to videos made by physicians or “other” (*P* = .403).

Of the 50 videos analyzed, 13 (26%) were YouTube “shorts.” YouTube “shorts” scored significantly lower on the CFR measure than the nonshort counterparts (0.8 vs. 4.3, *P* = .005). Differences in video power index, JAMA Benchmark Criteria, and video comprehensibility were not significant when comparing “shorts” to normal YouTube videos (Table VII).

**Table VII:** Mean values and standard deviations for video power index, JAMA Benchmark Criteria, video comprehensibility, and total CFR score, divided by video type (YouTube “short” vs. regular YouTube video).

Characteristic	Regular videos (N = 37)∗	YouTube “shorts” N = 13∗	*P* value†
Video power index	166 (457)	78,369 (201,241)	.224
JAMA Benchmark Criteria	1.32 (0.63)	1.08 (0.28)	.170
Video comprehensibility	3.38 (0.95)	3.62 (1.12)	.143
CFR score	4.3 (4.4)	0.8 (1.0)	**.005**

*JAMA*, Journal of the American Medical Association; *SD*, standard deviation.

Bold indicates statistically significant *P* values.

∗ Mean (SD).

† Wilcoxon rank sum test.

## Discussion

This study demonstrates that YouTube videos addressing clavicle fractures are generally incomplete, inconsistently reliable, and variable in educational value. These findings mirror trends reported across orthopedic subspecialties, where online videos often lack transparency, adequate sourcing, and clinically relevant detail.^1^^,^^5^^,^^7^^,^^14^^,^^17^^,^^27^^,^^32^^,^^35^ Prior analyses of online information for musculoskeletal disorders have similarly emphasized the overall low quality of unsupervised internet resources.^25^^,^^26^

## Educational completeness

CFR scores were low across all video categories (median: 3/18). Videos rarely addressed essential elements such as fracture classification, imaging interpretation, operative vs. nonoperative decision-making, and postoperative expectations—core principles supported by clavicle epidemiology and treatment literature.^3^^,^^8^^,^^10^^,^^12^^,^^13^^,^^15^^,^^16^^,^^21^^,^^23^^,^^28^^,^^30^^,^^33^^,^^38^ The absence of comprehensive information may contribute to patient misconceptions, unrealistic recovery expectations, and difficulty navigating treatment discussions with clinicians.

YouTube shorts were particularly deficient, scoring significantly lower on CFR compared with standard-length videos. This likely reflects limitations of short-form content described in prior digital media research.^19^^,^^22^ Because clavicle fractures commonly affect younger, digitally active patients,^6^^,^^10^^,^^23^ short-form content deficiencies are especially relevant.

### Reliability

Although physician-generated videos had higher JAMA scores than patient-generated videos, overall reliability remained low. Few videos included disclosure statements, source citations, or authorship—elements emphasized in the JAMA Benchmark Criteria.^25^^,^^29^ Even academically affiliated videos often lacked comprehensive attribution, echoing findings from prior assessments of online orthopedic education.^1^^,^^14^^,^^17^^,^^27^^,^^32^

The mean JAMA score of 1.4 is consistent with prior studies showing that orthopedic YouTube videos, whether related to knee arthroplasty, carpal tunnel syndrome, rotator cuff disease, or patellar instability, tend to score poorly on reliability metrics.^5^^,^^7^^,^^14^^,^^17^^,^^32^^,^^35^ Reviews of general medical information on the internet similarly highlight widespread deficits in transparency and governance.^25^^,^^26^

### Comprehensibility

Comprehensibility scores were relatively high, particularly among patient-generated videos, which often use simpler language and experiential descriptions. Although accessibility is beneficial, prior work has shown that such videos may sacrifice accuracy and completeness for relatability.^14^^,^^17^^,^^32^ Academic and physician-generated content tends to be more technically oriented, which may reduce perceived accessibility despite offering better reliability.^1^^,^^14^^,^^32^

The disconnect between comprehensibility and educational completeness mirrors patterns found in other orthopedic conditions, where patient-oriented videos are easier to understand but less medically informative.^17^^,^^27^^,^^32^ For clavicle fractures, where detailed discussions of classification, displacement, nonunion risk, and treatment algorithms are important,^3^^,^^8^^,^^12^^,^^16^^,^^21^^,^^30^ this gap represents a meaningful clinical concern.

## Clinical implications

Clavicle fractures frequently affect active, internet-reliant populations and involve treatment decisions that must be individualized.^6^^,^^10^^,^^15^^,^^16^^,^^21^^,^^23^ Meta-analyses comparing operative and nonoperative treatment show nuanced differences in union rates, recovery timelines, and functional outcomes.^4^^,^^9^^,^^18^^,^^33^^,^^36^^,^^37^ Without accurate online resources, patients may misunderstand the factors that influence decision-making, including displacement thresholds, patient activity level, and expectations for recovery.^3^^,^^8^^,^^16^^,^^21^^,^^30^

Given YouTube's global reach and high health-information traffic,^34^ improving the quality of online educational material represents an important opportunity. Studies across orthopedics and sports medicine increasingly advocate for clinician-directed, evidence-based, patient-friendly video content to counteract misinformation and enhance patient counseling.^1^^,^^7^^,^^14^^,^^17^^,^^27^^,^^32^^,^^35^ Orthopedic surgeons and academic institutions have an important role in this effort, particularly in developing accurate and accessible digital educational resources tailored to everyday patient needs. Such content should emphasize clear, nontechnical language, visual demonstrations of injury and rehabilitation concepts, transparent sourcing, and concise yet comprehensive explanations. Additionally, clinicians can improve digital literacy by educating patients on the limitations of online health information and encouraging critical evaluation of non–peer-reviewed resources.

### Limitations

This study has several limitations. YouTube content is dynamic, algorithm-dependent, and heavily influenced by viewer behavior, geographic variability, and emerging trends.^19^^,^^22^ Findings therefore represent a snapshot in time. The focus on the most viewed videos may exclude newer or less popular content that is higher in quality.

Scoring systems such as the JAMA Benchmark Criteria and CFR measure transparency and completeness but do not directly assess clinical accuracy, paralleling limitations recognized in prior video-quality research.^1^^,^^5^^,^^7^^,^^17^^,^^32^ Additionally, the CFR was a novel, author-developed scoring system that has not undergone external validation, which may limit its generalizability. While reviewer calibration improved scoring consistency, subjective interpretation may still influence results. Consolidation of low-frequency video categories, though necessary for statistical analysis, may also obscure granular differences among smaller subgroups.^17^^,^^27^

## Conclusion

YouTube videos related to clavicle fractures demonstrate generally low reliability and poor educational completeness despite favorable comprehensibility. A clear disconnect exists between accessibility and accuracy, with patient-generated content being easier to understand but less reliable and physician-generated content more reliable but less comprehensible; findings are consistent with prior analyses of online orthopedic information.^1^^,^^7^^,^^14^^,^^17^^,^^27^^,^^32^ High-quality, accurate, and engaging educational resources are needed to support patient understanding, enhance shared decision-making, and reduce the influence of misinformation.

## Disclaimers:

Funding: This research received no external funding. Kyle Masato Ishikawa is partially supported by the 10.13039/100006545National Institute on Minority Health and Health Disparities (grant number U54MD007601) and 10.13039/100000057National Institute of General Medical Sciences, 10.13039/100000002National Institutes of Health (grant number U54GM138062). The content is solely the responsibility of the authors and does not necessarily represent the official views of the NIH.

Conflicts of interest: The authors, their immediate families, and any research foundations with which they are affiliated have not received any financial payments or other benefits from any commercial entity related to the subject of this article.

## Declaration of generative AI and AI-assisted technologies in the writing process

Generative AI tools were not used in the preparation of this manuscript.
